# Role of viral coinfection in post-pandemic invasive Group A streptococcal infections in adults, a nation-wide cohort study (iGASWISS)

**DOI:** 10.1007/s10096-025-05229-y

**Published:** 2025-08-05

**Authors:** S. Musumeci, A. MacPhail, M. Weisser Rohacek, A. Erba, D. Goldenberger, C. Lang, E. Colin-Benoit, S. L. Leib, P. Sendi, C. Chuard, V. Erard, C. Fournier, L. Cobuccio, F. Tagini, C. Bertelli, L. Senn, R. Sommerstein, G. Blättler, C. Vieira Gomes, M. Moraz, S. Emonet, C. Strahm, P. Kohler, S. N. Seiffert, K. Herzog, D. Vuichard Gysin, S. D. Brugger, O. Nolte, A. Egli, D. Weller, A. M. Andrianaki, A. S. Zinkernagel, D-L Vu, A. Uribe Caparros, G. Laurie, N. Gaia, V. Lazarevic, P. François, J. Schrenzel

**Affiliations:** 1 Department of Infectious Diseases, Department of Diagnostics, University Hospital Geneva, Geneva, Switzerland; 2 Department of Infectious Diseases, Monash Health, Melbourne, Australia; 3 Division of Infectious Diseases, University Hospital Basel, Basel, Switzerland; 4 Clinical Bacteriology and Mycology, University Hospital Basel, Basel, Switzerland; 5 Department of Infectious Diseases, University Hospital Bern, University of Bern, Bern, Switzerland; 6 Institute for Infectious Diseases, University of Bern, Bern, Switzerland; 7 Unit of Infectious Diseases and Laboratory of Clinical Microbiology, Cantonal Hospital of Fribourg, Fribourg, Switzerland; 8 Department of Medicine, Division of Infectious Diseases, University Hospital Lausanne, Lausanne, Switzerland; 9 Institute of Microbiology, Lausanne University Hospital, Lausanne, Switzerland; 10 Faculty of Healthcare Sciences and Medicine, University of Lucerne and Klinik St. Anna, Lucerne, Switzerland; 11 Department of Infectious Diseases, Central Institute, Hospital of Valais, Valais, Switzerland; 12 Faculty of Medicine, Geneva, Switzerland; 13 Division of Infectious Diseases, Infection Prevention and Travel Medicine, Health Ostschweiz, Cantonal Hospital of St Gallen, St Gallen, Switzerland; 14 Division of Human Microbiology, Center for Laboratory Medicine, St. Gallen, Switzerland; 15 Department of Infectious Diseases, Institute of Laboratory Medicine, Spital Thurgau AG, Thurgau, Switzerland; 16 Department of Infectious Diseases and Hospital Epidemiology, University Hospital Zurich, University of Zurich, Zurich, Switzerland; 17 Institute of Medical Microbiology, University of Zurich, Zurich, Switzerland; 18 OCS (Office Cantonal de la Santé) of the Geneva Canton, Geneva, Switzerland

**Keywords:** Invasive group a *Streptococcus* (iGAS), Viral coinfection, *emm*1, M1UK clone, Post-pandemic epidemiology

## Abstract

**Objective:**

During the winter season of 2022–2023, numerous countries experienced a surge in invasive *Streptococcus pyogenes* (iGAS) infections. The role of viral coinfections in the post-COVID surge has not been elucidated. We report nation-wide data describing clinical presentation, microbiological characteristics, and associations with viral infection in adults during this period.

**Methods:**

A multicenter retrospective cohort study was conducted across 10 hospitals in Switzerland, including adults (> 16 years old) with iGAS infection from November 2022 to February 2023. Descriptive analysis was performed. A multivariable logistic regression model was fitted to assess the impact of viral coinfection. In addition, genetic analysis was performed in available isolates.

**Results:**

A total of 194 patients were included, with a median age of 50 years (interquartile range [IQR]: 37–69). 17/194 (8.8%) were immunosuppressed and 40/194 (20.6%) exhibited concomitant viral infections, predominantly Influenza A (21/40, 53%). Illness severity was high: 65/194 (33.5%) of cases necessitated admission to an intensive care unit (ICU), and the 30-day mortality was 4% (*n* = 8). Among the available strains for genetic analysis (*n* = 48), heterogeneity was found although ST28-*emm*1 isolates (also known as M1UK) was predominant (22/48).

**Conclusion:**

The post-COVID iGAS surge in Switzerland was associated with high levels of morbidity and mortality in immunocompetent adults. M1UK was predominant within the iGAS strains circulating in Switzerland during the study period and viral coinfection was a predictor for ICU admission and mortality.

**Supplementary Information:**

The online version contains supplementary material available at 10.1007/s10096-025-05229-y.

## Introduction

*Streptococcus pyogenes* (GAS), a Gram-positive, β-hemolytic *Streptococcus*, can cause infections ranging from mild localized to life-threatening invasive disease (iGAS) [1]. iGAS is defined as isolation of GAS from a sterile site or a severe clinical condition such as necrotizing soft tissue infection or streptococcal toxic shock syndrome (STSS) [2].

Throughout the winter season 2022–2023 several countries reported a substantial increase in iGAS cases in children and adults [3, 4] and different hypotheses have been raised to explain this. These include decreased GAS exposure, particularly among children during the COVID-19 pandemic leading to lower levels of immunity, the so called “immune debt” [5–8]; increased incidence of respiratory viruses related infections (Respiratory syncytial virus (RSV), Influenza, SARS-CoV-2) in the post pandemic period leading to an increase of bacterial superinfections [9]; or the potential presence of a more virulent clone [4].

The viral coinfection hypothesis also correlates in other bacterial infections. For instance, Rybak et al. [10]. described a decreased incidence of invasive pneumococcal diseases during COVID-19 pandemic, probably also associated with decreasing cases of RSV and Influenza, despite stable rates of pneumococcal carriage. Asymptomatic carriage of GAS is common, raising the possibility that GAS may have followed a similar pattern.

More generally, however, the association between respiratory viruses and GAS infections has not been fully established. Some studies highlighted an ecological temporal association between Influenza A virus and GAS [4, 11, 12]. Lung biopsies from the 1918 influenza pandemic revealed that both *S. pneumoniae* and GAS contributed substantially to the deaths of approximately 50 million people [13] and during 2010-2011, an increased iGAS incidence was observed in the United Kingdom (UK) during a high influenza season [13, 14]. Nevertheless, the contribution of respiratory viruses to iGAS seasonality or severity remains to be better defined.

In contrast, diversity of GAS isolates during the 2022–2023 winter surge suggests against a virulent clone as the main driver. Alcolea-Medina et al. [15]. in the UK found that the genes *emm*12 and *emm*1 represented 75% of the involved strains; *emm*1 was overrepresented in invasive cases with 80% related to M1UK clone. However, in The Netherlands, *emm* 1,4,12,22,89 accounted for more than 80% cases (all ages combined) [4] and in France the distribution was similar to the pre-pandemic period [9].

In Switzerland, iGAS is not a notifiable infectious disease, and there are limited data describing the post-pandemic epidemiology. The Swiss paediatric infectious disease group has described a steep increase in cases in children, but little is known on the epidemiology in adult population [16]. Preliminary data suggested an increase in bacteraemia cases during winter 2022-2023 [17, 18].

The iGASWISS study aimed to analyse nation-wide, clinical and outcome data for iGAS infection in adults in Switzerland during winter season 2022–2023. Specifically, we aimed to describe risk factors and outcomes, including the role of viral coinfection in the post-pandemic surge.

## Methods

### Inclusion criteria

We included patients older than 16 years old, with a diagnosis of iGAS infection between November 1, 2022 and February 28, 2023, hospitalized in one of 10 participating hospitals in Switzerland (University Hospitals of Geneva, Lausanne, Bern, Basel, Zürich; Cantonal Hospital of St Gallen, Valais, Fribourg, Thurgau and the Hirslanden Klinik St Anna in Luzern). Collectively these hospitals service a catchment area of around 5 million people. iGAS was defined as a laboratory isolation of GAS from any normally sterile site, or isolation from a non-sterile site in a patient with necrotizing tissue infection or streptococcal toxic shock syndrome.

### Microbiological techniques

GAS was identified through standard culture techniques or polymerase chain reaction (PCR). Available strains (*n* = 48) were genotyped using a dedicated in-house developed MLVA assay (Supplementary material S1). Emm-typing was performed using CDC procedure (https://www.cdc.gov/strep-lab/php/group-a-strep/emm-typing.html) and confirmed using blast on assembled genomes sequenced by using Illumina sequencer (Supplementary material S1). MLST (multilocus sequence typing) was deduced in silico from assembled whole genome sequences.

### Data collection

Data was collected retrospectively from the electronic medical records by participating centres via REDCap^®^ (Research Electronic Data Capture) database.

### Statistical analysis

Descriptive analysis was performed of patient demographics (age, gender, canton of diagnosis, presence of immunosuppression, travel history, concomitant viral infection), iGAS presentation (site[s] of infection, presence of bacteraemia, presence of shock), treatment (administration of antitoxin therapy, administration of intravenous immunoglobulin [IVIg], surgery), and outcomes (requirement for intensive care unit [ICU], mechanical ventilation [MV], extra corporeal membrane oxygenation [ECMO] and death at 30 days).

Immunosuppression was defined as HIV infection with CD4 < 100/µl, malignancy, cytotoxic chemotherapy, solid organ transplantation, receipt of immunosuppression therapies including biologics, antimetabolites, or small molecule inhibitors.

To explore risk factors for ICU admission and/or death, including the role of viral coinfection, a logistic regression model was fitted to calculate risk of a composite endpoint of death at 30 days or admission to intensive care. This composite endpoint was chosen due to low overall number of deaths. Model variables were selected based purely on clinical significance and included: age, sex, and immunosuppression. Formal assessment of collinearity was made by considering variance inflation factor (VIF) with all included variables reporting VIF < 5, and model plausibility validated by assessment and comparison of univariable and multivariable effect estimates. Analyses were performed using STATA v18 (Statacorp, Texas, USA).

### Ethical approval

This study was approved by the Ethics Committee of Geneva (Switzerland) with the agreement of the Ethics Committees of Zurich, Bern, Vaud, Nordwest-Central and East Switzerland (BASEC-ID: 2023-00623). Informed consent was sought from participating patients. Patients declining to sign a general consent or any other declining statement against using data for research purposes were not included.

## Results

### Patients' characteristics

We identified 194 cases of iGAS during the four-month study period. Patient characteristics are reported in Table 1. A near-equal distribution of male (50.5%, n=98) and female (49.5%, n=96) patients was found, with a median age of 50 years old (IQR: 37-69). Only 6.2% (n = 12) reported a recent travel history within the preceding four weeks, 8.8% (n= 17) had an immunosuppressive condition. The most reported sites of infection were bacteraemia (alone or concomitant with other conditions, 40.2%, n = 78) followed by abscess (35.6%, n= 69)

**Table 1:** Clinical characteristics and outcomes of iGAS infection in Switzerland in 2022–2023 winter surge

Patients characteristics (*n* = 194)		*n*	(%)
Male		98	(50.5)
Female		96	(49.5)
Age median (years)		50	(IQR : 37–69)
Recent travel history (up to 4 weeks before)		12	(6.2)
Immunosuppression		17	(8.8)
Associated viral infection		40	(20.6)
Clinical presentation			
Bacteraemia (alone or associated with other conditions)		78	(40.2)
Abscess		69	(35.6)
Shock (Septic/Toxic)		43	(22.1)
Pneumonia		33	(17)
Necrotizing tissue infection		26	(13.4)
Empyema		12	(6.2)
Osteoarticular infection		21	(10.8)
Meningitis		5	(2.6)
Endocarditis		2	(1.0)
Others*		11	(5.6)
Treatment			
Any dedicated surgery (at least 1)		102	(52.6)
IVIg (%, n)		16	(8.2)
Receipt of antitoxin treatment**		65	(33.5)
Outcomes			
ICU admission		65	(33.5)
* ICU median days*		*7*	*(IQR 3–15)*
Mechanical ventilation		40	(20.6)
ECMO		7	(3.6)
30-day mortality		8	(4.1)

* *Others* includes pelvic inflammatory disease (PID)/endometritis (*n* = 3), mediastinitis (*n* = 2), septic bursitis (*n* = 2), septic thrombophlebitis (*n* = 2), and unknown site (*n* = 2)

** *Antitoxin treatments* refer to clindamycin and/or linezolid

### Emm typing and MLVA

**Fig. 1:**
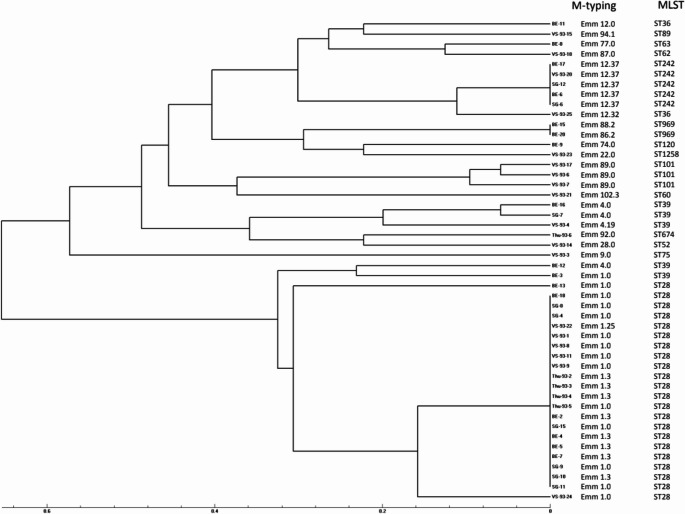
Available strains (*n* = 48) genotyped via a dedicated in-house developed MLVA assay. Genotyping tree using multilocus variable-number tandem repeat analysis (MLVA) of representative isolates identified in the different Swiss Cantons. The main cluster contains 40% of strains of the collection and shows isolates harboring *emm*1 belonging to sequence type 28, originating from 4 different Cantons. The scale indicates the level of genetic relatedness within this set of strains based on UPGMA analysis.

Genotyping assay depicted on Fig. 1, shows diversity of genetic backgrounds. Among the 48 characterized isolates, *emm*1 types were overrepresented (n=23) and co-clustered within a large cluster of Sequence Type (ST) 28. Three other small clusters are visible containing *emm*4 (n=3) *emm*89 (n=3) and *emm*12 types (n=6) representing the same sequence type, ST39, ST101 and ST242, respectively.

Whole genome sequence information used for comparative genomics purposes (Supplementary material S2) shows pairwise single nucleotide polymorphism (SNP) comparison analysis and reveals a limited distance between all strains from all Cantons. The largest number of SNPs between isolates was <150 suggesting a limited distance between strains. Absence of any SNP difference was frequently observed for pairs of strains isolated in the same Canton but also between Cantons. The four clusters containing the prevalent genotypes noticed above, revealed very high homogeneity with SNP numbers generally <5.

### Treatment

52.6% of patients (n = 102) required at least one dedicated surgical intervention. Antibiotics with anti-toxin effect (either clindamycin or linezolid) were administered in one third of patients (33.5%, n=65) and intravenous immunoglobulins in 8.2 % (n=16).

### Outcomes

One third of patients (33.5%, n=65) required admission to an ICU. Among these, mechanical ventilation was required in 61.5% (n=40), and extracorporeal membrane oxygenation (ECMO) in 10.8% (n = 7). Median ICU length of stay was 12 days [IQR 3-15]. Among patients requiring ICU admission, 47.7% were female (n = 31) and 16.9% had an immunosuppressive condition (n = 11).

There were eight deaths at 30 days, including two without ICU admission. Median age of patients who died was 65 years old (IQR: 58-87); 6/8 were female; 1/8 had a concomitant immunosuppressive condition; and 4/8 died within 48 hours from admission.

### Presence of viral coinfection

**Fig. 2:**
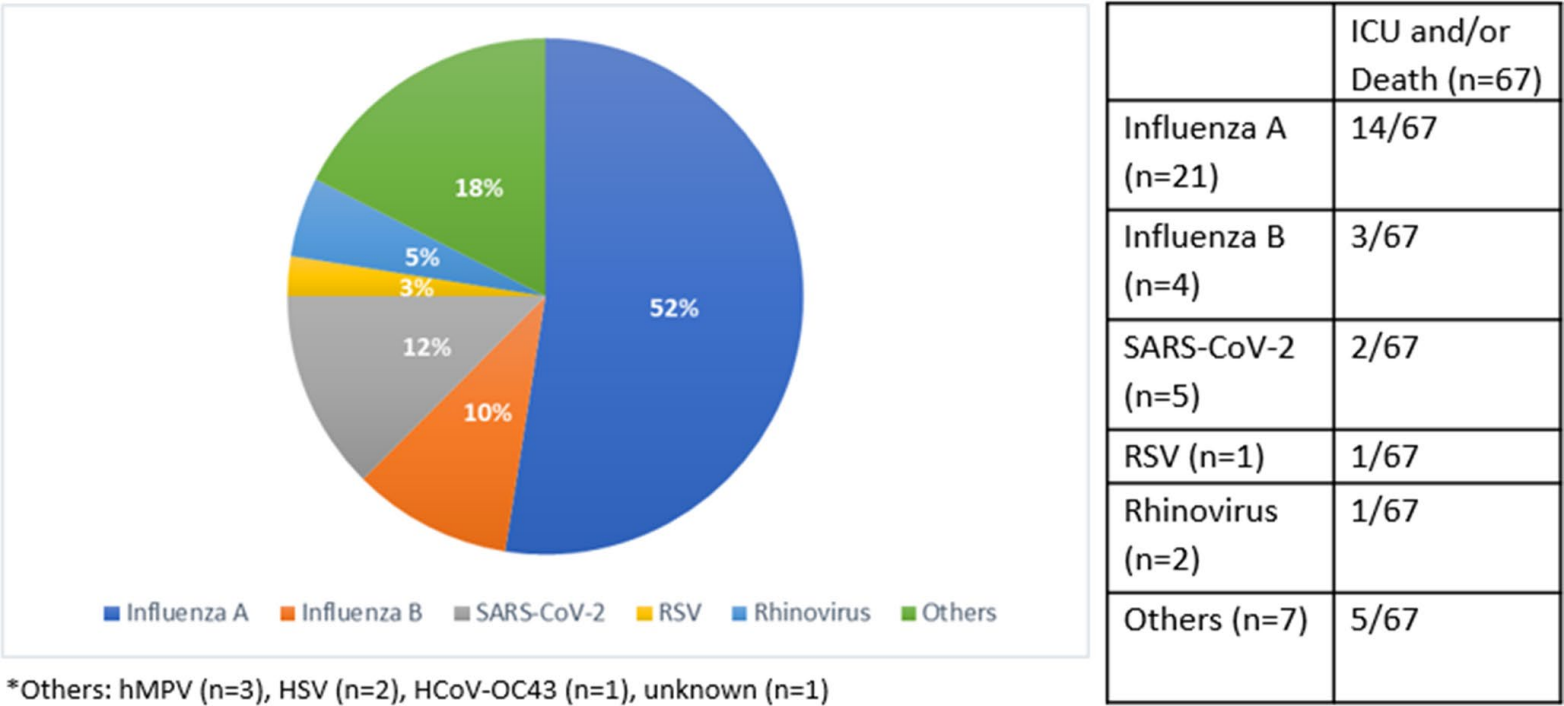
Respiratory viral coinfections in iGAS cases and associated severity outcomes (ICU admission and/or death). The pie chart illustrates the proportional distribution of detected respiratory viruses among iGAS patients with any viral coinfection. The table shows the number of patients with each virus. The left column indicates the total number of iGAS cases associated with each detected virus. The right column displays the subset of those patients who experienced a severe outcome, defined as ICU admission and/or 30-day mortality.

20.6% (n=40) of iGAS cases were associated with concomitant viral infection, most commonly Influenza A (21/40 cases) (Fig. 2). Among patients who died or required ICU admission, viral coinfection was present in 26/67 (38.8%). Among patients who died, 4/8 had a concomitant viral infection (3/4 Influenza A virus).

### Risk factors for ICU admission or death

A total of 67/194 (34.5%) patients either required ICU admission or died within 30 days. Risk factors for ICU admission and/or death are described in Table 2.

**Table 2:** Associations with ICU admission and death

Variable of interest	Composite Mortality and ICU admission		Univariable			Multivariable		
	n	(%)	OR	95% CI	*P*	OR	95% CI	*P*
Overall	67/194	(34)	Reference			Reference		
Male	34/98	(35)	1.01	(0.56–1.83)	0.96	1.08	(0.57–2.08)	0.81
Age > 65	27/61	(43)	1.84	(0.98–3.45)	0.06	1.74	(0.88–3.46)	0.11
Any immunosuppression	11/17	(62)	3.9	(1.39–11.25)	0.01	4.84	(1.60–14.61)	0.01
Viral coinfection	26/40	(65)	5.12	(2.43–10.74)	< 0.01	5.63	(2.61–12.16)	< 0.01

Model AUROC = 0.73

Composite outcome refers to either ICU admission or 30-day mortality

*OR* Odds ratio; *CI *Confidence interval

Multivariable model adjusted for age, sex, immunosuppression, and viral coinfection

The composite outcome of mortality and ICU admission was significantly higher in patients aged over 65, those who were immunosuppressed, and those with viral coinfection.

Viral coinfection appeared as an independent predictor of the composite endpoint of ICU admission or mortality in the multivariable model (OR 5.63 95% CI 2.61. 12.16).

Given that Influenza A accounted for more than half of all cases of viral coinfection, we performed sensitivity analysis first including only cases of Influenza A, and then excluding Influenza A. In both models, viral coinfection remained significant (*p* <0.01).

Clinical characteristics of patients with or without ICU admission or death are reported in supplementary Table 1. Bacteraemia, necrotising infection and shock were the most frequent presentations resulting in ICU or death, while abscess and empyema were less frequently associated with adverse outcomes.

Given the previous relationship between *emm*1 subtype and invasive infection, we performed a supplementary analysis of those patients with genotyping available (n = 48), stratified by *emm*1 subtype vs other [19]. Patient characteristics and outcomes of patients with or without *emm*1 subtype are reported in supplementary Table 2. Patients with infection caused by *emm*1 were significantly older (median age 65 years, IQR 43-78 vs median age 41 years (IQR 32-52) and more likely to have bacteraemia (65% versus 32%). There was no significant difference in other sites of infection, immunosuppression, travel, presence of viral coinfection, or outcomes including mortality and ICU admission.

## Discussion

We performed a nationwide retrospective cohort study of adult patients with iGAS infection in Switzerland during the 2022-2023 winter surge and identified 194 cases. Most cases occurred in immunocompetent adults, of relatively young age (median 50 years). Morbidity was high, with one third requiring intensive care unit admission and 30-day mortality reached 4%. Viral coinfection, particularly Influenza A, was an independent risk factor for ICU admission and death.

This is an important finding. In the setting of shifting viral epidemiology in the post-pandemic period, it was not known to what extent co-infection represented a driving factor in iGAS infections. Our findings suggest that improved coverage of vaccine-preventable viral infections may also potentially reduce the risk and consequences of iGAS infections. Alternate theories, such as the “immune debt” hypothesis, may also be contributory.

Among the available strains for genetic analysis (n=48), heterogeneity was found although ST28- *emm*1 (also known as M1UK) was overrepresented (22/48; 45.8%). Recent national and international surveillance data support our findings. The UK Health Security Agency (UKHSA) and other European networks reported a marked increase in iGAS cases during the 2022–2023 winter season, particularly among children and young adults, with a predominance of *emm*1 and the M1UK clone, a known hypervirulent lineage associated with increased toxin production and enhanced invasiveness [15, 20]. Elsewhere, Bellés-Bellés A et al. recently described the *emm*49 as a potential emergent cause in iGAS cases in Spain, raising attention on the importance of continuous surveillance in different geographic areas [21]. Among patients with genetic analysis available, those with infections caused by *emm*1 subtypes were older than patients with other subtypes, and more likely to have bacteraemia. We did not detect any differences in clinical profile and outcome; however, this analysis may have been underpowered as genetic analysis was only available for a minority of patients.

The interaction between respiratory viruses and invasive GAS is biologically plausible and has been described in several studies. Viral infections, especially Influenza A, can predispose to bacterial superinfections through mucosal barrier disruption, upregulation of bacterial adhesion receptors, and modulation of innate immunity—including impaired neutrophil function and altered cytokine responses [11, 22]. These mechanisms may facilitate systemic invasion by hypervirulent GAS clones such as M1UK, thereby compounding disease severity.

The *emm* typing and multiple-locus VNTR analysis (MLVA) performed on the available strains, while having less discriminatory power than the whole genome sequencing (WGS) provided a fast, simple, and cost-effective method for outbreak investigations by giving valuable insights into the genetic diversity and epidemiology of the *Streptococcus pyogenes* and sets a comparison baseline for future investigations.

In Switzerland, as iGAS infections are not subject to compulsory declaration, there are few previous data describing characteristics of these infections. To our knowledge this is the largest multicentre study ever performed on iGAS infections in this country. Participating centres ranging from large tertiary care hospitals to private clinics in different areas of the country encompassed a diverse patient population, mitigating potential selection bias and enhancing generalizability.

However, our study has several limitations. The retrospective study design relied upon medical records review and increases the risk of bias. Only hospitalized patients across the ten participating sites were included, which may lead to an underrepresentation of milder cases. Nevertheless, due to the severity of the iGAS definition itself, and the large catchment area included in the study, we think this is unlikely to have substantially influenced out findings. Detailed comorbidity data beyond immunosuppressive conditions such as diabetes, cardiovascular and renal disease, trauma, and postpartum status were not systematically collected, which may have limited our ability to fully characterize patient susceptibility and severity determinants. Due to the retrospective nature of the study and the structure of the case report form, differentiation between septic shock and streptococcal toxic shock syndrome (STSS) was not consistently possible. Toxin profiling was not performed, and thus we cannot confirm how many patients with reported shock fulfilled formal STSS criteria.

Finally, the short study period did not allow longitudinal analysis or comparison to the pre-pandemic period.

In conclusion, the winter 2022-2023 iGAS surge in Switzerland was associated with significant morbidity in adults. Viral coinfection appears to be a mediating factor in the illness severity and M1UK clones were overrepresented.

Continuous surveillance and collaboration between clinicians, researchers and public health authorities remain necessary to further elucidating the underlying mechanism and better prepare for current and future global health challenges.

## Supplementary Information

Below is the link to the electronic supplementary material.

Supplementary Material 1

Supplementary Material 2

Supplementary Material 3

## Data Availability

No datasets were generated or analysed during the current study.
