# Supplementary material for: Role of viral coinfection in post-pandemic invasive Group A streptococcal infections in adults, a nation-wide cohort study (iGASWISS)
Source: Eur J Clin Microbiol Infect Dis. 2025 Aug 5;44(11):2677–84. doi: 10.1007/s10096-025-05229-y (PMC12619775; doi:10.1007/s10096-025-05229-y)
Supplement: Supplementary file 1 — Supplementary Material 1 [file 10096_2025_5229_MOESM1_ESM.docx]

**Supplementary material S1**

**Genotyping MLVA assay**

The general methodology has been described in the past by our group for several bacterial species [1-3]. Repeat-containing target genes were selected by scanning the whole genomes of reference strains available in public NCBI database [4]. A consensus sequence was then identified for each gene by using the BLAST alignment (<http://www.ncbi.nlm.nih.gov/BLAST/>). After localization of the region of interest, primers were selected by using Jellyfish (version 1.3; Biowire). Particular attention was paid to the amplicon size. Nucleic acids from the three reference strains were simultaneously assayed with the 20 clinical isolates, which were used as positive controls (data not shown). Conditions for amplification were as follows: first step (t1), 2 min at 95°C; t2, 15 s at 95°C; t3, 20 s at 60°C; t4, 40 s at 72°C (t2 to t4, repeated 35 times); and t5, 10 min at 74°C. The total volume of each PCR mixture (KOD Hot Start; Novagen) was 20 μl and contained 0.8 U of KOD polymerase and all primers listed in Table 1 at the indicated concentrations. After PCR amplification, 1 μl of each PCR mixture was loaded on a DNA-7500 chip and run on the BioAnalyzer Agilent 2100 instrument. Expert application was used to monitor the overall quality of the experiments. The variability of the PCR and the amplicon migration time were evaluated by using DNA from reference isolate. The PCR was performed in triplicate with four independent DNA extraction products (12 samples). Each PCR amplification product was randomly loaded four times on four BioAnalyzer chips. The complete protocol was performed three times with independent samples. This experiment was used to define the cutoff values that could be used to distinguish related from unrelated strains; 0.1.

BioAnalyzer output flat files (.csv) containing raw fluorescence data were exported into specifically developed software. The fluorescence data were rescaled according to their ladder positions to allow comparison of the results for multiple samples. The relative distance between the rescaled data was assessed by cross-correlation coefficient calculation [1], which consists of comparison of the fluorescence values between samples at each time point. The results were subjected to hierarchical clustering as previously described [1].

**DNA purification and sequencing**

DNA was purified from single colonies grown on blood agar plates using DNeasy columns (Qiagen). High-throughput sequencing was used to sequence the genomes of 48 strains using the Illumina NovaSeq 6000 (Illumina, San Diego, California), using 150 base pairs (bp) paired-end reads and bar codes strategy according to the DNA-seq strategy following the manufacturer's recommendations. Read quality was assessed with the Fastqc program (available at: <http://www.bioinformatics.babraham.ac.uk/projects/fastqc/>) and filtered using TRIMMOMATIC v0.39 (available at: <http://www.usadellab.org/cms/?page=trimmomatic>). Genome assembly was performed using Spades v3.15.5 [5] with following parameters: -k 21,33,55,77,99 --careful. Assembled genomes were annotated using the Prokka v1.10 program [4]. Multilocus sequence typing analysis was performed using annotated genomes and submitted to the Center for Genomic Epidemiology database (available at: <http://cge.cbs.dtu.dk/services/MLST>). The phylogenetic relationship of all isolates was investigated by genomic single-nucleotide polymorphism (SNP)–based analysis using CSI Phylogeny using standard parameters for assessment of SNPs quality and Z-score [6]. The genome of *Streptococcus pneumonia* NCTC7465 was used as reference genome.

The Blastn algorithm was used to confirm *Emm*-type obtained by Sanger sequencing.

**Gentoyping MLVA assay primers**

>Spy1_MLVA_F

AGTTGCGGTGTCAGCATCAG

>Spy1_MLVA_R

AGCGCAGCAGCTGTAAAGAA

>Spy2_MLVA_F

CAGCTGCTGAAGATGGCTTATCAG

>Spy2_MLVA_R

GCGTTGGGGTAACGAGTATAGC

>Spy3_MLVA_F

AGGTGCAAGTGCGGTTAAGG

>Spy3_MLVA_R

GTCGTGAGCTGCCGGTGTTTTTG

>Spy4_MLVA_F

CCTGATCACTATTAGTAACAGACTTAAC

>Spy4_MLVA_R

ACTGCATGATGACTGGGTACAC

>Spy5_MLVA_F

GCCAACTAAGGGTTCAGGTCAG

>Spy5_MLVA_R

TCTGGTTCCTTGTTATCAAAGTGGA

>Spy6_MLVA_F

CTCACGTAAGCCGCTGATGA

>Spy6_MLVA_R

CTCCCAAAGACCAGTCGTCTC

>Spy7_MLVA_F

TAAATTCTCAAACATCTTATCAGTTTCATTTTGAG

>Spy7_MLVA_R

CTCAAACAACTGATGATGCTGACAGAGACTATG

>VNTR_GAS_8F

AACATCTAAAGCGRTTGGAGTT

>VNTR_GAS_8R

AAAGTGAAATGCTTTTTGCGTA

>VNTR_GAS_9F

ACCTAAAAGAACGAGACCAAGC

>VNTR_GAS_9R

GTTTTGCAACAGCTACAACTCC

>VNTR_GAS_10F

GGTACAAACAAACCGAAACGTA

>VNTR_GAS_10R

ATACGCATTCAAAACGTTCATC

**References used in the supplementary section S1**

1. Francois, P.; Huyghe, A.; Charbonnier, Y.; Bento, M.; Herzig, S.; Topolski, I.; Fleury, B.; Lew, D.; Vaudaux, P.; Harbarth, S.; et al. Use of an automated multiple-locus, variable-number tandem repeat-based method for rapid and high-throughput genotyping of Staphylococcus aureus isolates. *Journal of Clinical Microbiology* **2005**, *43*, 3346-3355.

2. Francois, P.; Hochmann, A.; Huyghe, A.; Bonetti, E.J.; Renzi, G.; Harbarth, S.; Klingenberg, C.; Pittet, D.; Schrenzel, J. Rapid and high-throughput genotyping of Staphylococcus epidermidis isolates by automated multilocus variable-number of tandem repeats: a tool for real-time epidemiology. *J.Microbiol.Methods* **2008**, *72*, 296-305.

3. Cavanagh, J.P.; Klingenberg, C.; Hanssen, A.M.; Fredheim, E.A.; Francois, P.; Schrenzel, J.; Flaegstad, T.; Sollid, J.E. Core genome conservation of Staphylococcus haemolyticus limits sequence based population structure analysis. *J.Microbiol.Methods* **2012**, *89*, 159-166.

4. Benson, G. Tandem repeats finder: a program to analyze DNA sequences. *Nucleic Acids Res.* **1999**, *27*, 573-580.

5. Bankevich, A.; Nurk, S.; Antipov, D.; Gurevich, A.A.; Dvorkin, M.; Kulikov, A.S.; Lesin, V.M.; Nikolenko, S.I.; Pham, S.; Prjibelski, A.D.; et al. SPAdes: a new genome assembly algorithm and its applications to single-cell sequencing. *J Comput Biol* **2012**, *19*, 455-477, doi:10.1089/cmb.2012.0021.

6. Kaas, R.S.; Leekitcharoenphon, P.; Aarestrup, F.M.; Lund, O. Solving the problem of comparing whole bacterial genomes across different sequencing platforms. *PLoS One* **2014**, *9*, e104984, doi:10.1371/journal.pone.0104984.
