# Supplementary material for: Role of viral coinfection in post-pandemic invasive Group A streptococcal infections in adults, a nation-wide cohort study (iGASWISS)
Source: Eur J Clin Microbiol Infect Dis. 2025 Aug 5;44(11):2677–84. doi: 10.1007/s10096-025-05229-y (PMC12619775; doi:10.1007/s10096-025-05229-y)
Supplement: Supplementary file 3 — Supplementary Material 3 [file 10096_2025_5229_MOESM3_ESM.docx]

**Supplementary table 1: Comparison of patient characteristics with or without adverse outcome**

|  | Patient characteristics (n=194) | Adverse outcome (n = 67) | | No adverse outcome (n = 127) | |  |
| --- | --- | --- | --- | --- | --- | --- |
|  |  | n | (%) | n | (%) | p |
| Male | | 34 | (51%) | 64 | (50%) | 0.966 |
| Female | | 33 | (49%) | 63 | (50%) |  |
| Age median (years) | | 60 | (IQR 40-73) | 47 | (IQR 36-66) | 0.03 |
| Recent travel history (up to 4 weeks before) | | 2 | (3%) | 10 | (8%) | 0.18 |
| Immunosuppression | | 11 | (16%) | 6 | (5%) | <0.01 |
| Associated viral infection | | 26 | (39%) | 14 | (11%) | <0.01 |
| Clinical presentation | |  |  |  |  |  |
|  | Bacteraemia (alone or associated with other conditions) | 40 | (60%) | 38 | (30%) | <0.01 |
|  | Abscess | 6 | (9%) | 63 | (50%) | <0.01 |
|  | Shock (Septic/Toxic) | 40 | (60%) | 3 | (2%) | <0.01 |
|  | Pneumonia | 17 | (25%) | 16 | (13%) | 0.02 |
|  | Necrotizing tissue infection | 21 | (31%) | 5 | (4%) | <0.01 |
|  | Empyema | 8 | (12%) | 4 | (3%) | 0.02 |
|  | Osteoarticular infection | 5 | (8%) | 16 | (13%) | 0.27 |
|  | Meningitis | 3 | (5%) | 2 | (2%) | 0.23 |
|  | Endocarditis | 2 | (3%) | 0 | (0%) | 0.05 |
|  | Others | 2 | (3%) | 9 | (7%) | 0.24 |
| Treatment | |  |  |  |  |  |
|  | Any dedicated surgery (at least 1) | 41 | (61%) | 61 | (48%) | 0.08 |
|  | IVIg (%, n) | 16 | (24%) | 0 | (0%) | <0.01 |
|  | Receipt of antitoxin treatment***** | 49 | (73%) | 16 | (13%) | <0.01 |

Adverse outcome = composite of admission to intensive care or death within 30 days. * Antitoxin treatments: clindamycin and/or linezolid. Comparisons made using chi2 test.

**Supplementary table 2: Patient characteristics and outcomes of patients with or without *emm*1 subtype**

|  | Patient characteristics  (n=48) | *emm*1 (n = 23) | | Other *emm* type (n = 25) | |  |
| --- | --- | --- | --- | --- | --- | --- |
|  |  | n | (%) | n | (%) | p |
| Male | | 14 | (61%) | 8 | (32%) | 0.05 |
| Female | | 9 | (39%) | 17 | (68%) |  |
| Age median (years) | | 65 | (IQR 43-78) | 41 | (IQR 32-52) | <0.01 |
| Recent travel history (up to 4 weeks before) | | 0 | (0%) | 3 | (12%) | 0.09 |
| Immunosuppression | | 2 | (9%) | 2 | (8%) | 0.93 |
| Associated viral infection | | 6 | (26%) | 3 | (12%) | 0.21 |
| Clinical presentation | |  |  |  |  |  |
|  | Bacteraemia (alone or associated with other conditions) | 15 | (65%) | 8 | (32%) | 0.02 |
|  | Abscess | 3 | (13%) | 7 | (28%) | 0.20 |
|  | Shock (Septic/Toxic) | 8 | (35%) | 6 | (24%) | 0.41 |
|  | Pneumonia | 2 | (9%) | 3 | (12%) | 0.71 |
|  | Necrotizing tissue infection | 4 | (17%) | 5 | (20%) | 0.82 |
|  | Empyema | 1 | (4%) | 1 | (4%) | 0.95 |
|  | Osteoarticular infection | 3 | (13%) | 3 | (12%) | 0.91 |
|  | Meningitis | 2 | (9%) | 0 | (0%) | 0.13 |
|  | Endocarditis | 0 | (0%) | 0 | (0%) | N/A |
|  | Others | 1 | (4%) | 4 | (16%) | 0.19 |
| Treatment | |  |  |  |  |  |
|  | Any dedicated surgery (at least 1) | 11 | (48%) | 17 | (68%) | 0.16 |
|  | IVIg (%, n) | 3 | (13%) | 1 | (4%) | 0.26 |
|  | Receipt of antitoxin treatment***** | 10 | (43%) | 10 | (40%) | 0.81 |
| Outcomes | |  |  |  |  |  |
|  | ICU admission | 11 | (48%) | 7 | (28%) | 0.16 |
|  | ICU median days | 3 | (IQR 1-15) | 14 | (IQR 1-23) | 0.38 |
|  | Mechanical ventilation | 5 | (22%) | 5 | (20%) | 0.88 |
|  | ECMO | 0 | (0%) | 2 | (8%) | 0.17 |
|  | 30-day mortality | 4 | (17%) | 2 | (8%) | 0.33 |

* Antitoxin treatments: clindamycin and/or linezolid. Comparisons made using chi2 test
